# Primary hyperparathyroidism: targeted, focused exploration with “selective” parathyroidectomy: Minimally invasive open vs. minimally invasive video-assisted vs. endoscopic approach

**DOI:** 10.1007/s00508-026-02703-1

**Published:** 2026-02-02

**Authors:** Lindsay Hargitai, Philipp Riss, Christian Scheuba

**Affiliations:** Division of Visceral Surgery, Department of General Surgery, Medical University of Vienna, Waehringer Guertel 18–20, Vienna, Austria

**Keywords:** Sporadic primary hyperparathyroidism, Bilateral neck exploration, Focused parathyroidectomy by minimally invasive access, Preoperative localization, Intraoperative parathyroid hormone monitoring

## Abstract

Sporadic primary hyperparathyroidism is most commonly caused by a single parathyroid adenoma. Traditionally, bilateral neck exploration with assessment of all four glands was the gold standard, achieving cure rates up to 98%. This approach has largely been replaced by limited exploration (LE), in which a prelocalized hyperfunctioning gland is selectively removed using a small open, video-assisted, or endoscopic approach.

Successful LE relies on accurate preoperative localization of single-gland disease. First-line imaging consists of high-resolution ultrasound combined with 99mTc-sestamibi scintigraphy with single-Photon Emission Computerized Tomograph (SPECT/CT). When results are negative or discordant, 18F-choline PET/CT is recommended, significantly improving localization and enabling targeted surgery.

.Because multiglandular disease cannot be definitively excluded preoperatively, intraoperative parathyroid hormone (IOPTH) monitoring has become an important adjunct. IOPTH confirms complete excision of hyperfunctioning tissue and aids detection of additional abnormal glands. Several interpretive criteria exist, including Miami, Vienna, Halle, and Rome, with Miami and Vienna most commonly used. Although routine IOPTH use in concordantly localized single-gland disease remains debated, evidence suggests it reduces persistent disease and reoperation rates, particularly when imaging is inconclusive.

Endoscopic, extracervical, and robotic approaches offer superior cosmetic outcomes but involve greater dissection, higher costs, and increased technical demands, limiting widespread use. Overall, advances in imaging and intraoperative adjuncts have enabled minimally invasive parathyroidectomy to replace bilateral exploration while maintaining excellent long-term outcomes.

## Introduction

Sporadic primary hyperparathyroidism (PHPT) is caused by a single parathyroid adenoma (single-gland disease, SGD) in the majority of patients. Selective surgical excision (selective parathyroidectomy, SP) of the enlarged, hyperfunctioning gland remains the only curative treatment option with long-term success rates of up to 98% at initial surgery [1, 2].

Due to a lack of alternative treatment options up until the 1990s, the standard surgical treatment for PHPT was extended bilateral neck exploration (BNE) by a standard (around 50 mm) skin incision, during which all four parathyroid glands were systematically identified and any visibly macroscopically enlarged gland(s) were removed. Efforts to develop less extended approaches were limited for a long time by difficulties in accurately localizing enlarged, hyperfunctioning parathyroids preoperatively and predicting cure intraoperatively with high accuracy after removing the prelocalized hyperfunctioning single gland. [3].

Over the last two decades the development of efficient localization studies [4] has fundamentally changed the surgical management of PHPT from extended (BNE) to limited neck exploration (LE). These improvements have enabled a targeted, focused exploration with image-guided selective removal of a single parathyroid adenoma. All surgical approaches to SP (with the skin incision in the neck) are considered minimally invasive (minimally invasive parathyroidectomy, MIP) and can be performed (mini-incision) open, video-assisted or (complete) endoscopically (with/without robotic device) [3]. The use of MIP also enables access to the ipsilateral gland if required. The advantages of LE with SP over BNE include reduced costs, shorter operating times, better cosmetic outcomes with higher patient satisfaction and patients may be treated on an outpatient basis with at least identical complication rates [5]. Furthermore, MIP has a success rate of 95–97%, similar to classical BNE [2, 5]. Thus, LE with any of the available MIP techniques has largely replaced BNE in patients with preoperatively well-localized single parathyroid adenoma. Recently, extracervical (remote access) approaches (via the retro-auricular, axillary, unilateral/bilateral supramamillary or by transoral endoscopic exploration via a vestibular route) are endoscopically applied in single highly specialized centers [6]; however, conventional BNE with IOPTH continues to be the standard approach in complex cases, for example when sporadic multiglandular disease (MGD) is suspected in localization studies in the hereditary variants of PHPT (which are always in connection with MGD) or when preoperative localization is inconclusive or negative [3].

## Localization techniques

Preoperative localization is performed after biochemical diagnosis of PHPT. Its primary aim is to identify the enlarged, hyperfunctioning parathyroid glands, including any that are ectopic and to plan and definitively guide the operative approach [7].

### Ultrasound

The most common generally available, first-line morphological examination includes high-resolution real-time ultrasound (US). The standard examination is performed with the patient’s neck in slight extension, using a high-frequency linear probe (7.5–15 MHz) in both transverse and longitudinal planes giving the size of the lesion in three dimensions. Particular attention is directed to the area posterior to the thyroid and medial to the carotid and jugular vessels, where the parathyroid glands are typically found [8]. The examination should extend from the carotid bifurcation down to the sternal notch, should include the upper thoracic outlet with the thyrothymic ligaments (if possible), should include the paratracheal regions and the carotid-jugular corridor [9]. Any thyroid lesion has to be carefully documented not to miss concomitant thyroid nodules necessitating additional thyroid surgery [10] or intrathyroidal ectopic or supernumerary parathyroid tumors [11].

The US examination demonstrates a positive localization result with an overall accuracy of 88% and a positive predictive value (PPV) of 78–97% [12, 13]. Enlarged glands are typically described as hypoechoic, well-circumscribed lesions with hyperechoic margins and are usually ovoid in shape [14]. The advantages of US include noninvasiveness, affordability, rapidity and no radiation; however, its sensitivity can be affected by multiple parathyroid adenomas, parathyroid hyperplasia or concurrent thyroid nodules [15, 16]. Furthermore, its accuracy is operator-dependent and differentiation between lymph nodes and parathyroid adenomas can pose challenges [17]. Endocrine surgeons who can perform their own US can possibly increase the accuracy of parathyroid adenoma identification [18].

Combining US with functional imaging, such as 99mTc-sestamibi scintigraphy (MIBI) improves performance [7, 19].

### ^99m^Tc-sestamibi scintigraphy

The use of 99mTc-sestamibi (MIBI) scintigraphy is the gold standard functional imaging modality for preoperative localization of parathyroid tumors in PHPT. It emerged as a successful parathyroid imaging tool following initial explorations with ^201^thallium chloride in combination with ^99m^Tc-pertechnetae as dual-tracer imaging in the 1980s [20]. The first report on the use of ^99m^Tc-MIBI in parathyroid imaging was in 1989 [21].The ^99m^Tc-MIBI is a lipophilic cationic compound that concentrates in hyperfunctioning parathyroid glands due to the increased number of mitochondria, which is primarily due to a higher proportion of oxyphilic cells [22]. Dual-phase imaging (early phase 10–30 min and delayed phase 90–150 min) is conducted after injection [23]. Given that The ^99m^Tc-MIBI has a greater uptake per gram of parathyroid tissue compared to the thyroid tissue, as well as a slower washout from the hyperactive parathyroid and these properties make it a good tracer for parathyroid imaging [24]. The MIBI scintigraphy can be performed as planar or tomographic single-photon emission computed tomography (SPECT) combined with computerized tomography (CT) imaging (SPECT/CT). This offers additional anatomical information, which further increases the diagnostic accuracy [14] and enables minimally invasive access to the upper or lower enlarged glands to be planned in more detail. Several meta-analyses have verified the superior diagnostic accuracy of SPECT and SPECT/CT over planar imaging [25, 26]. Reported sensitivities range from 56–100%, with overall accuracy approaching 97% [13, 27, 28]. Combining US and MIBI scintigraphy further improves sensitivity [13]. False positives most commonly arise from solid thyroid nodules [29] and can decrease the sensitivity of MIBI by 15–39% [30]. Delayed tracer washout may occur in thyroid carcinoma, reactive lymph nodes, metastatic lymphadenopathy, PTH-secreting paragangliomas and hypertrophic submandibular salivary glands [30, 31]. False negative findings most often occur in small adenomas or in MGD [29, 31]. Even multimodal imaging cannot fully exclude MGD [13]. Reduced accuracy in parathyroid hyperplasia may reflect the absence of mitochondrial DNA mutations that drive oxyphilic cell proliferation in parathyroid adenomas [32]. Continued advances in imaging resolution are expected to further refine diagnostic performance.

### Four-dimensional computerized tomography

The cross-sectional image applying four-dimensional computerized tomography (4D-CT) in PHPT was first described by Rodgers et al. in 2006 [33]. Parathyroid adenomas are characterized by their hypodensity on noncontrast imaging, intense arterial enhancement and rapid venous washout, distinguishing them from thyroid tissue and lymph nodes [34]. Once used primarily in re-operative settings, 4D-CT is increasingly employed as a first-line or second-line modality when US or MIBI scintigraphy results are inconclusive.

Its major limitation is radiation exposure: the effective dose averages 10.4 mSv compared with 7.8 mSv for SPECT/sestamibi imaging [35], and the thyroid dose is substantially higher (92 mGy compared to 1.6 mGy with sestamibi), translating to an estimated 0.1% risk of thyroid malignancy [35]. Its use is further restricted in patients with renal insufficiency or iodinated contrast agent allergies.

The use of 4D-CT shows high localization sensitivity in SGD (88–92%) [23, 36], outperforming US (57%) and MIBI scintigraphy (65%) in one series [35], but sensitivity significantly decreases in MGD (43–69%) [37, 38].

### Positron emission tomography/computerized tomography

Positron emission tomography (PET) provides high-resolution functional imaging through intravenous radiotracer administration. When fused with CT, it offers precise anatomical localization [39]. For parathyroid PET/CT imaging, choline can be labelled with a positron-emitting isotope, most commonly ^11^C‑choline or ^18^F‑choline (FCH). Choline is a vital nutrient required for the synthesis of phospholipids, key structural elements of cell membranes. Its cellular uptake rises when choline kinase is upregulated. Tissues with rapid membrane turnover, such as tumor or adenoma cells, demand increased amounts of choline to support their heightened phospholipid production [40]. Hyperfunctioning parathyroid glands were first identified incidentally in prostate cancer patients undergoing FCH PET/CT as part of their cancer assessment [41].

Because of its short half-life (20.3 min), ^11^C‑choline PET/CT requires on-site cyclotron production, which is not possible for many locations [14]. More suitable for clinical imaging is ^18^F‑choline PET/CT, which has a longer half-life of 110 min [14]. Several studies and meta-analyses report high sensitivity up to 95% and PPV of 97% for ^18^F‑choline PET/CT, outperforming ^11^C‑choline PET/CT [42–44]. PET/CT offers shorter execution times, higher efficiency and lower radiation dose (6 mSv versus 8 mSv) with higher resolution imaging [42, 45]. Furthermore, PET/CT is the best preoperative imaging modality for identifying ectopic adenomas and is less operator dependent than US [45]. False positives may arise from mediastinal lymph nodes, thyroid nodules and chronic autoimmune thyroiditis, while false negatives occur in borderline biochemical disease and MGD [23, 46]. Limitations include tracer availability, the number of PET centers as well as cost, although wider adoption and longer tracer half-life are improving accessibility and cost-effectiveness [47]. Overall, PET/CT is typically more costly than conventional scintigraphy and SPECT/CT. These expenses, however, may be reduced when an on-site cyclotron is available or when the institution serves as a high-volume referral center. Moreover, PET/CT costs are expected to continue declining as newer scanners and improved software enable lower tracer doses and PET-CT is increasingly recognized as a valuable first-line option, particularly when MGD is suspected [45].

### Practical advice

Accurate preoperative localization enables targeted, focused SP with a mini-incision open technique or applying any of the available video-assisted or endoscopic techniques. Once biochemical confirmation of hyperparathyroidism is established, standard practice entails localization studies with US and MIBI scintigraphy. Concordant results support a LE (with IOPTH), whereas discordant or negative findings typically prompt BNE with IOPTH monitoring [13, 48].

When available, ^18^F‑choline PET-CT is recommended when either initial modality is negative, allowing many patients to proceed with targeted surgery should the PET-CT offer a positive localization. Advanced imaging has been shown to be more cost-effective when compared to standard BNE in patients with nonlocalized PHPT [49].

Because no modality achieves perfect localization and performance varies by operator and institutional volume [25], IOPTH monitoring remains essential to exclude MGD, even when imaging suggests single-gland involvement [50, 51].

## The role of intraoperative parathyroid hormone monitoring

The precise identification of hyperfunctional parathyroid glands remains challenging, largely because 3–6% of individuals exhibit embryological positional variation and the number of glands may vary [52]. Focused exploration with SP risk missing a second ipsilateral or contralateral parathyroid tumor(s), which may lead to persistent hyperparathyroidism. Intraoperative parathyroid hormone monitoring (IOPTH) acts as a valuable adjunct during parathyroid surgery, confirming cure or identifying residual hyperfunctioning tissue after removal of the diseased gland. Because parathyroid hormone (PTH) has a short half-life (2–3 min) [53], levels drop rapidly following removal of a solitary adenoma permitting real-time confirmation of a biochemical cure. With the development of a rapid PTH assay [54], IOPTH was first introduced by Nussbaum et al. in 1988 [55] and then in 1991 by Irvin et al. [56] and has enabled targeted SP in patients with well-localized parathyroid adenomas. A sufficient intraoperative drop in PTH confirms a cure, while further exploration is required if PTH fails to adequately decline, suggesting MGD [57]. The necessity of IOPTH in clearly localized SGD remains debated [58, 59]. According to the German Association of Endocrine Surgeons (Surgical Working Group Endocrinology, CAEK), surgeons can omit IOPTH when imaging is concordant for SGD [60]. Other studies recommend that IOPTH should still be used even in concordantly localized SGD [61, 62]. Likewise, the American Association of Endocrine Surgeons advocates its continued use to avoid operative failure [7].

Several intraoperative criteria to document a sufficient drop in PTH levels as a predictor of cure have been defined. The Miami criterion, introduced by Irvin et al. [63, 64], is a widely acknowledged and commonly utilized intraoperative protocol. An operation is considered successful when a reduction of ≥ 50% in PTH from either the highest baseline or pre-excision hormone level, measured 10 min after excision of all hypersecreting glands. A recently published meta-analysis determined the sensitivity of the Miami criterion to lie between 95–97% and specificity 56–78% [65]. Surgical cure is confirmed when normocalcemia persists for a minimum of 6 months postoperatively.

In a study by Riss et al. [50], the Vienna criterion was introduced. It mandates obtaining a baseline preincision PTH sample before beginning the surgery. A reduction of ≥ 50% from the baseline PTH level at 10 min post-gland resection indicates a successful operation [50]. Previous studies have determined the sensitivity of the Vienna criterion to lie between 89.1–92%, specificity 69.4–88.5% and overall accuracy 88–91.6% [50, 66, 67]. In a recently published study it was demonstrated that in patients with low basal PTH values (< 100 pg/ml), other criteria may be better suited for this patient population [67]. Caution also needs to be taken with intraoperative PTH spikes when using the Vienna criterion, as studies have shown that PTH spikes can lead to a higher rate of interpretational errors [50, 68, 69], which was also confirmed by a recently published study [66]. One of the most important components of the Vienna criterion is its high probability of diagnosing MGD intraoperatively. This is due to its strict limits, which result in higher specificity than other criteria; however, it can also lead to a higher rate of conversions to unilateral neck exploration (UNE) or BNE when compared to the Miami criterion, due to its lower sensitivity [66].

The Halle criterion stipulates that PTH should fall intraoperatively within the low normal range (≤ 35 ng/L) within 15 min of removing all hyperfunctioning parathyroid glands [70]. In the literature, the sensitivity of the Halle criterion lies between 62.9–69.8%, specificity 88.6–100%, and overall accuracy 65–71.9% [50, 71].

Based on a study [72] that highlighted the importance of a 20-min endpoint for improved identification of MGD and preventing surgical failures, the Rome criterion was introduced in 2008 by Lombardi et al. [73]. The Rome criterion necessitates a reduction of ≥ 50% from the highest pre-excision PTH level and/or a PTH level within the reference range at 20 min following excision and/or a PTH level at least 7.5 ng/L lower than the level recorded at 10 min post-excision [73]. This criterion is more geared to patients with MGD as it demonstrates a 90% specificity in identifying MGD when compared to the Miami criterion with only 50% specificity; however, it has a higher false negative (FN) rate than the Miami criterion (16% versus 5%) [73]. In the literature, the sensitivity of the Rome criterion lies between 82.9–83.3%, specificity 90–100%, and overall accuracy 83.6–83.8% [71, 73].

Comparative studies show that stricter criteria improve MGD identification but increase false negatives, leading to unnecessary BNE [63, 71]. Barczynski et al. [71] evaluated the accuracy of each criterion in predicting operative success in 260 patients with PHPT. Barczynski et al. [71] reported the highest accuracy with the Miami criterion (97%), followed by Vienna (92%), Rome (84%), and Halle (65%) criteria, whereas other studies have shown superior MGD performance with Vienna and Halle criteria [50]. Furthermore, the overall accuracy was similar between the Miami (93%) and the Vienna (92%) criteria [50]. In the study by Barczynski et al. [71] the Miami criterion yielded overall the most favorable results as an adjunct to parathyroid surgery, with sensitivity, specificity, positive predictive value (PPV) and negative predicative value (NPV) of 97.6%, 93.3%, 99.6% and 70%, respectively; however, one must consider that only patients with SGD and not MGD were included in this study. Furthermore, while Barczynski et al. [71] concluded that the Vienna criterion is not better than other criteria in terms of predicting postoperative normocalcemia due to a false positive (FP) rate of 0.4%, other studies contradict this conclusion [74]. In a recently published study, the employment of the Vienna criterion intraoperatively predicted normocalcemia in 98–100% of patients, which was confirmed in up to 99% of patients after 12 months follow-up [67]. Table 1 lists all the commonly used criteria.

**Table 1:** Various IOPTH criteria and their accuracy (adapted from Barczynski et al. [71])

Criterion	Sensitivity (%)	Specificity (%)	PPV (%)	NPV (%)	Accuracy (%)
Miami	97.6	93.3	99.6	70	97.3
Vienna	92.2	93.3	99.6	60.9	92.3
Halle	62.9	100	100	14.2	65
Rome	82.9	100	100	26.3	83.8

*IOPTH* intraoperative parathyroid hormone monitoring; *PPV* positive predictive value; *NPV* negative predictive value

Although no single IOPTH criterion is universally applicable for hyperparathyroidism, multiple studies have shown that the employment of IOPTH  can prevent reoperations in approximately 3–9.6% of patients [59, 75, 76] while achieving a success rate of 97–99% in sporadic PHPT [77, 78]. The use of SP performed without IOPTH carries a higher failure rate of 1–2.7% [58, 79]. While some [61, 62] have reported that IOPTH remains necessary even in patients with concordantly localized SGD, other studies have questioned its routine use in such cases, noting that it can increase operative time, costs and conversion rates when intraoperative PTH decline is insufficient [58, 59, 79, 80].

### Practical advice

The use of IOPTH has enabled accurately localized parathyroid adenomas to be treated with (targeted, focused) SP shifting the surgical paradigm away from BNE. By reducing reoperation rates it helps avoid the additional risks and costs of secondary procedures, postoperative complications and the management of persistent hypercalcemia. It remains essential in patients with discordant or negative preoperative imaging, where the likelihood of MGD is highest. Importantly, persistence rates increase by over 7% when IOPTH is omitted, even in suspected localized SGD, underscoring its continued diagnostic value [62]. Thus, the continued use of IOPTH as state of the art, in accordance with the recommendation of the American Association of Endocrine Surgeons [7] is advised.

## Surgical techniques in PHPT

For years BNE with selective removal of the parathyroid gland(s) assessed as macroscopically pathological was the standard operation for proven PHPT.

As documented [81, 82], the procedure is associated with very low mortality (0.2%) and modest morbidity, including wound infection (0.8%), postoperative hemorrhage (1.5%) and recurrent laryngeal nerve injury (0.5%). In less experienced hands, the risk of long-term hypoparathyroidism is higher. Persistent hypocalcemia is directly related to the extent of exploration and gland excision and occurs less frequently with a focused surgical approach [81].

### Practical advice

Following the introduction of IOPTH in 1991 and the advances in preoperative localization over the past two decades, a re-evaluation of the traditional BNE approach has been prompted. As a single adenoma accounts for at least 85% of cases, a scan-directed LE is sufficient for most patients and avoids unnecessary bilateral dissection. Since 2000, LE techniques have gained popularity, achieving success rates comparable to BNE while reducing postoperative complications, shortening hospital stays, and improving cosmetic outcomes [78, 83].

## Open minimally invasive parathyroidectomy (OMIP)

Open SP by a mini-cervical incision is called open minimally invasive parathyroidectomy (OMIP) and has since gained widespread popularity [84]. The procedure is performed through a Kocher incision of approximately 20–30 mm positioned about a fingerbreadth above the sternal notch in the midline or paramedian. The single enlarged adenoma is exposed without endoscopic equipment and excised using retractors. Theoretically a UNE can be performed and the incision can be extended to BNE if conversion becomes necessary.

### Practical advice

Compared with BNE, LE with mini-incision and SP (OMIP) is feasible everywhere and offers several advantages, including shorter operation times, reduced costs, suitability as an outpatient procedure and is less traumatic [2, 5, 48]. Its success rate of 95–98% is comparable to that of conventional BNE [5, 48]. Evidence from high-volume centers indicates no significant differences in complication rates, costs or operation time [85], whereas a meta-analysis demonstrated a lower rate of recurrent laryngeal nerve injury and postoperative hypocalcemia comparing LE and BNE [2]. In centers with limited experience, OMIP is recommended when preoperative imaging identifies a parathyroid adenoma as the gland can be excised without exploring the remaining parathyroids and adequacy of resection can be confirmed using IOPTH monitoring. Consequently, LE applying minimal access techniques have largely replaced BNE in patients with localized disease. Classical BNE remains the procedure of choice when preoperative imaging fails to localize the presumed affected gland or when primary hyperplasia or hereditary disease (always involving all parathyroid tissue) is suspected or preoperatively confirmed [5].

## Minimally Invasive Video-assisted Parathyroidectomy (MIVAP)

The video-assisted gasless technique (minimally invasive video-assisted parathyroidectomy, MIVAP) employs a 20-mm midline incision above the sternal notch [86]. Visualization is achieved using a thin endoscope and small retractors, with the strap muscles retracted to expose the adenoma. Contralateral exploration can be performed without extending the incision and reported conversion rates in experienced centers are approximately 8%.

The primary advantages of this technique include direct access to the affected gland and the use of small cervical incisions. Studies also described reduced postoperative pain, improved cosmetic outcomes, shorter hospital stays and the feasibility of performing these procedures as an outpatient compared to traditional parathyroidectomy [87, 88]. Nonetheless, because the incisions are still located on the neck (1.5cm transverse incision positioned 2 cm above the sternal notch), they remain visible and can be susceptible to hypertrophic or keloid scarring. A recent meta-analysis comparing MIVAP to OMIP [89] demonstrated that OMIP was associated with lower conversion rates and was not inferior to MIVAP in any secondary outcomes except for length of stay and scar length, suggesting that OMIP may represent the overall more favorable technique. In addition, the cost-effectiveness of OMIP remains a key advantage, as MIVAP requires specialized endoscopic training and equipment [89]; however, due to hypertrophic or keloid scarring, Asian surgeons started exploring extracervical techniques.

## Endoscopic parathyroidectomy

### Anterior endoscopic approach

First described by Gagner in 1996 [90], this fully endoscopic technique is performed under continuous CO_2_ insufflation. A 5‑mm endoscope is introduced through a central trocar, with two or three additional trocars used for instrumentation. Dissection begins in the subplatysmal plane to create an adequate working space, after which the midline is opened, and the strap muscles are retracted to expose the thyroid lobe. The parathyroid glands are then identified following separation of the thyroid from the surrounding fascia. This approach offers excellent cosmetic outcomes due to its small, remote access incisions; however, it is technically demanding and challenging to replicate, particularly for surgeons without dedicated endoscopic expertise. Additionally, risks associated with CO_2_ insufflation cannot be entirely avoided. Although effective with minimal morbidity in experienced centers, this technique has not gained widespread adoption but has served as a stepping stone for the development of further endoscopic approaches.

### Lateral endoscopic approach

First described by Henry et al. [91], this technique involves a 12-mm skin incision along the anterior border of the sternocleidomastoid muscle (SCM), positioned 3–4 cm above the sternal notch on the side of the affected parathyroid gland. Through this incision, tissue is dissected using an open technique to reach the prevertebral fascia. Once an adequate working space is created, two additional 2.5-mm trocars are placed along the anterior border of the SCM, 3–4 cm above and below the initial incision, and a 10-mm trocar for a 0° endoscope is inserted. Dissection is performed under continuous CO_2_ insufflation at 8 mm Hg throughout the procedure. The primary technical limitation of this approach is its unilateral access, which restricts the ability to perform a bilateral exploration without converting to a conventional open procedure.

### Extracervical/remote access approaches

Remote access exploration techniques place all incisions necessary to explore the parathyroids outside the neck and require extensive subcutaneous dissection. The working space is maintained either through CO_2_ insufflation or with specialized external retractors. First performed without a robotic device the procedures now integrate robotic aid.

The first robotic-assisted parathyroidectomy was described by Tolley et al. [92] and subsequent integration of robotic technology has further facilitated remote access parathyroid surgery, in some cases eliminating the need for insufflation [93]. The da Vinci platform (Intuitive Surgical, Sunnyvale, CA, USA) can reduce the technical difficulty of certain extracervical approaches. Furthermore, by providing a high-definition, three-dimensional view and employing advanced robotic instruments that accurately mimic hand movements, the da Vinci system improves the surgical team’s safety and performance. These features ensure optimal visualization of vital structures, including the superior laryngeal nerve, the recurrent laryngeal nerve (RLN) and the parathyroid glands [94].

A recent review on robotic-assisted parathyroidectomy [95] reported no cases of RLN injury, postoperative hypoparathyroidism, or bleeding. All patients achieved a cure, although 6.2% required conversion to the conventional open approach [95]. Postoperative complications occurred in 9.2% of patients, all were managed conservatively and cosmetic outcomes were excellent in all cases [95]. Another study [96] reported improved long-term quality of life outcomes with robotic-assisted parathyroidectomy, based on visual analogue scales (VAS) and EuroQOL-5D scores, including significantly reduced postoperative pain and better cosmetic results up to 24 months.

The most widely conducted robotic-assisted approaches include transaxillary, bilateral axillo-breast approach (BABA) and the recently introduced single-port robotic areolar (SPRA) approach.

The transaxillary approach, first described in 2009 has reached a cumulative total of 5000 reported cases by 2018 [94, 97]. An axillary incision is performed followed by a wide dissection to reach the neck, calling into question its designation as minimally invasive [95]. Furthermore, the wide subcutaneous dissection can result in flap-related complications such as pain and paresthesia [98]. The most significant drawback, however, is the inability to perform bilateral exploration because contralateral exposure is inadequate [99]; however, this approach produces an excellent, hidden scar, leading to high patient satisfaction [93, 100].

While the transaxillary approach is limited to one side of the patient, BABA provides identical visualization of both thyroid lobes, allowing procedures without instrument changes. First implemented in 2008 [101] the BABA robotic approach offers the shortest learning curve, with four incisions in the bilateral axilla and breast that make the technique easier for surgeons to master. Further advantages include the familiar top-down view of open thyroidectomy. It also eliminates the need for retractors, reducing traction-related complications and cervical lymph node dissection, is feasible, as its oncological safety has been demonstrated [102]; however, it requires extensive subcutaneous tissue dissection to create the flap, extending from both axillae and breasts across the anterior chest to the neck. Paresthesia or pain may develop beneath the flap area, and the risk of seroma formation is higher following BABA surgery [103]. The thin flaps can predispose patients to postoperative adhesions [104]. To date, there has been only one study published on BABA parathyroidectomy versus open parathyroidectomy [105]. The authors demonstrated that the BABA robotic parathyroidectomy group consisted of younger patients and had a longer average operative time [105]. This suggests that younger patients place greater emphasis on cosmetic outcomes and are more inclined to select scarless surgical options [105]. Transient hypoparathyroidism was observed in two open surgery patients and one BABA robotic parathyroidectomy patient. Regardless of technique, all 74 patients attained biochemical cure within 6 months [105].

Following the launch of the da Vinci SP single port system in 2018, the newest robotic approach SPRA was recently introduced [106]. As a refinement of the BABA technique, SPRA eliminates bilateral axillary access and therefore requires less flap dissection, making it a less invasive option [106]. By limiting flap dissection to the region between the right breast and the neck, the SPRA approach eliminates the need for axillary access on both sides and results in a reduction of more than 50% in flap area [106]. After docking the da Vinci SP, the exploration proceeds similarly to the standard BABA approach. Flap creation takes less than 15 min, roughly half the time required for BABA [102, 106, 107]. It is currently only being used in thyroid surgery but also shows much potential for parathyroid surgery.

### Practical advice

While robotic surgical approaches are very appealing, they do have several limitations. The most significant is the markedly higher cost compared with conventional open parathyroidectomy, with total expenses potentially exceeding $ 1.5 million when factoring in the robotic platform, maintenance and case-specific equipment [96]. While robotic-assisted parathyroidectomy has been shown to be safe and free of major complications such as RLN injury or bleeding, many studies reported longer operative times than those observed with traditional cervical parathyroidectomy [96]. Robotic-assisted parathyroidectomy is also limited by the need for highly specialized training and certification in robotic surgery, restricting its adoption to experienced endocrine surgical teams and well-equipped hospitals [96, 100].

## Transoral endoscopic parathyroidectomy vestibular approach (TOEPVA)

The transoral endoscopic vestibular approach for thyroid (TOE*T*VA) and parathyroid (TOE*P*VA) surgery was first described by Anuwong in 2016 [108] and has since gained international interest due to its scarless access and favorable early outcomes. Although more than 860 TOETVA cases have been reported [109], published experience with TOEPVA remains limited. Recent reviews of TOEPVA demonstrated a 96% success rate and a 3.8% overall complication rate, including one transient recurrent laryngeal nerve injury (1.3%) comparable to outcomes from open procedures [109, 110]. No permanent complications occurred [110]. Infection risk appears lower than initially anticipated [111] and the absence of cutaneous incision eliminates wound-specific morbidity such as dehiscence or keloid formation. Furthermore, it should be recognized that the transoral vestibular approach may limit access to an enlarged superior parathyroid gland. Manipulation during extraction could result in glandular injury and subsequent parathyroid hormone leakage, producing a transient PTH spike that may affect intraoperative PTH measurements.

Patient selection varies due to the lack of standardized criteria; most institutions exclude individuals with prior neck surgery, thyroiditis, previous radiation therapy, non-localizing parathyroid tumor or suspected malignancy [111].

### Practical advice

Operative times for TOEPVA and other remote access approaches consistently exceed those of open parathyroidectomy [84, 109]. This likely reflects differences in surgeon experience and the technical demands of endoscopic dissection; however, reductions in operative duration are expected with increased procedural volume. Potential cost implications remain unclear and require further evaluation.

Compared with other remote approaches, TOEPVA has a shorter learning curve, facilitated by familiar cervical dissection planes and a direct route to bilateral parathyroid glands [112]. Its principal advantage is the absence of a visible cervical scar, an outcome shown to have meaningful psychosocial and cosmetic value particularly among younger and female patients [113, 114].

## Conclusion

Advances in imaging and development of alternative surgical techniques have fundamentally transformed the management of primary hyperparathyroidism, shifting practice from extended BNE toward LE performed through minimal access open, video-assisted or endoscopic techniques.

High-accuracy localization studies now enable the majority of patients with single-gland disease to undergo LE with targeted, focused SP with outcomes comparable to traditional more extended BNE, while offering similar complication rates and faster postoperative recovery. Monitoring of IOPTH remains a valuable tool, especially when imaging is inconclusive, as it confirms biochemical cure and aids in the detection of multiglandular disease.

An expanding range of endoscopic remote access techniques, including extracervical and transoral approaches with or without robotic assistance, provides excellent cosmetic results; however, their adoption is influenced by the need for advanced endoscopic expertise, increased procedural costs, and careful patient selection.

Ultimately, optimal treatment of primary hyperparathyroidism requires an individualized approach that integrates imaging quality, surgeon experience, and patient preference to ensure a safe procedure and long-lasting cure.
